# Epigenetic and epitranscriptomic regulation during oncogenic *γ*-herpesvirus infection

**DOI:** 10.3389/fmicb.2024.1484455

**Published:** 2025-01-07

**Authors:** Rajnish Kumar Singh, Ramakrishna Vangala, Atharva S. Torne, Dipayan Bose, Erle S. Robertson

**Affiliations:** Departments of Otorhinolaryngology-Head and Neck Surgery and Microbiology, Perelman School of Medicine, University of Pennsylvania, Philadelphia, PA, United States

**Keywords:** EBV, KSHV, epigenetics, epitranscriptomics, cancer

## Abstract

Oncogenic gamma herpesviruses, including Epstein–Barr Virus (EBV) and Kaposi’s Sarcoma-associated Herpesvirus (KSHV), are opportunistic cancer-causing viruses and induces oncogenesis through complex mechanisms, which involves manipulation of cellular physiology as well as epigenetic and epitranscriptomic reprogramming. In this review, we describe the intricate processes by which these viruses interact with the epigenetic machinery, leading to alterations in DNA methylation, histone modifications, and the involvement of non-coding RNAs. The key viral proteins such as EBNA1 and LMP1 encoded by EBV; LANA and vGPCR encoded by KSHV; play pivotal roles in these modifications by interacting with host factors, and dysregulating signaling pathways. The resultant reprogramming can lead to activation of oncogenes, silencing of tumor suppressor genes, and evasion of the immune response, which ultimately contributes to the oncogenic potential of these viruses. Furthermore, in this review, we explore current therapeutic strategies targeting these epigenetic alterations and discuss future directions for research and treatment. Through this comprehensive examination of the epigenetic and epitranscriptomic reprogramming mechanisms employed by oncogenic gamma herpesviruses, we aim to provide valuable insights into potential avenues for novel therapeutic interventions.

## Introduction

Gamma herpesviruses are a subfamily of the Herpesviridae family and are characterized by their ability to establish lifelong latent infections (Cohen, 2020). Infection with a number of these viruses shows strong association with various disease phenotypes (Cohen, 2020; Charostad et al., 2020). Epstein–Barr Virus (EBV) and Kaposi’s Sarcoma-associated Herpesvirus (KSHV) are the two prominent members of this subfamily. EBV is widely known for causing infectious mononucleosis and is implicated in the development of several malignancies, including Burkitt’s lymphoma, Hodgkin’s lymphoma, and nasopharyngeal carcinoma (Baumforth et al., 1999). KSHV infection, on the other hand, is the etiological agent of Kaposi’s sarcoma, primary effusion lymphoma, and multicentric Castleman’s disease (Giffin and Damania, 2014). Both viruses exhibit a dual lifecycle, alternating between latent and lytic phases, which allows them to persist in the host and evade immune detection. During latency, these viruses express a limited set of genes that can manipulate host cellular processes to create a favorable environment for viral persistence and oncogenesis (Uppal et al., 2015; Krishnan et al., 2004).

Epigenetic reprogramming is a fundamental process by which both EBV and KSHV persist successfully within the infected host cell and manipulate host cellular environments to promote oncogenesis (Chen et al., 2013). Unlike large scale genetic changes, epigenetic modifications alter gene expression without altering the underlying DNA, enabling these viruses to easily and reversibly influence cellular pathways (Hamilton, 2011; Pei et al., 2020). This epigenetic reprogramming allows these viruses to create a cellular environment that supports viral persistence and replication by silencing viral as well as antiviral host-encoded genes and activating pathways that promote cell survival and proliferation (Pei et al., 2020; Paschos and Allday, 2010). For instance, EBV can methylate the promoters of tumor suppressor genes, leading to their silencing, which removes critical restrictions to uncontrolled cell growth (Saha et al., 2015; Zhang L. et al., 2022). Similarly, KSHV utilizes its latent nuclear antigen (LANA) and other antigens such as vGPCR to recruit host epigenetic machinery (Toth et al., 2016; Singh et al., 2018). These changes results in the activation of oncogenes and the repression of immune response genes (Charostad et al., 2020). Additionally, epigenetic reprogramming plays a key role in the immune evasion strategies by these viruses (Locatelli and Faure-Dupuy, 2023). By modifying the expression of genes involved in antigen presentation, immune recognition and immune response, both EBV and KSHV can escape detection by the host immune system to facilitate the establishment of latency and genome persistence (Locatelli and Faure-Dupuy, 2023; White et al., 2010; Fiches et al., 2020). Persistent infection by both EBV and KSHV further allows the accumulation of epigenetic as well as genetic changes that can mediate malignant transformation. The reversible and dynamic nature of epigenetic changes also suggests that these modifications can be dynamically regulated in response to environmental factors, providing the viruses with the flexibility to adapt to different stages of infection and disease progression (Tempera and Lieberman, 2014; Scott, 2017). Both EBV and KSHV can mediate changes in the major pathways, and the mechanisms of epigenetic reprogramming that includes DNA methylation, histone modification and non-coding RNAs (Rehman et al., 2023; Srivastava et al., 2023). EBV and KSHV, significantly alter host DNA methylation patterns to promote oncogenesis (Journo et al., 2021; Matsusaka et al., 2017). DNA methylation typically occurs at the 5′ position of cytosine residues within CpG dinucleotides, leading to the formation of 5-methylcytosine. This modification generally represses gene expression (Moore et al., 2013). EBV employs several strategies to manipulate DNA methylation where EBV-encoded latent membrane protein 1 (LMP1) can upregulate DNA methyltransferases (DNMTs), the key enzymes involved in adding methyl groups to the DNA (Luo et al., 2018). This results in the hypermethylation and subsequent silencing of tumor suppressor genes such as p16INK4a and E-cadherin, facilitating uncontrolled cellular proliferation and metastasis (Burassakarn et al., 2017). Similarly, KSHV-encoded LANA interacts with DNMTs to promote the hypermethylation of tumor suppressor gene promoters, ensuring the maintenance of a proliferative and survival-promoting environment for infected cells (Shamay et al., 2006).

Histone modifications, the other crucial aspect of epigenetic reprogramming is also influenced by EBV/KSHV infection (Srivastava et al., 2023; Pietropaolo et al., 2021). These modifications include but not limited to methylation, acetylation, phosphorylation, ubiquitination, and sumoylation of histone proteins (Loboda et al., 2019; Li et al., 2014). These modifications can greatly affect chromatin structure as well as expression of associated genes (Van Opdenbosch et al., 2012). EBV and KSHV manipulate these modifications to favor viral persistence as well as oncogenesis by modulating expression of histone modifying enzymes. For example, EBV-encoded EBNA2 recruits histone acetyltransferases (HATs) to both viral and cellular promoters, leading to histone acetylation and transcriptional activation of genes involved in cell proliferation and survival, such as c-Myc and Cyclin D1 (Van Opdenbosch et al., 2012; Zhou et al., 2015). Conversely, KSHV-encoded LANA can influence histone deacetylases (HDACs) to deacetylate histones at specific promoters, leading to gene repression (Lu et al., 2014). Additionally, both EBV and KSHV can induce histone methylation changes. EBV can increase the levels of histone H3 lysine 27 trimethylation (H3K27me3) through the recruitment of the polycomb repressive complex 2 (PRC2), leading to silencing of genes that inhibit cell growth and survival (Dochnal et al., 2021).

In addition to direct modification of DNA or histone at the chromatin level, non-coding RNAs (including microRNAs and long non-coding RNAs) also play significant roles in epigenetic regulation through utilization of a trans regulatory strategy (Liu W. et al., 2020). Both EBV and KSHV encodes several miRNAs that can modulate both viral and host gene expression (Liu W. et al., 2020; Notarte et al., 2021). EBV-encoded miR-BART6 can downregulate the expression of the tumor suppressor gene DICER, which is involved in miRNA processing, thereby globally affecting miRNA-mediated gene regulation (He et al., 2016). KSHV-encoded miRNAs also target host genes regulating apoptosis, immune evasion, and angiogenesis (Samols et al., 2007). Furthermore, both EBV and KSHV can induce the expression of cellular miRNAs that favor oncogenesis (Vojtechova and Tachezy, 2018). lncRNAs mediated regulation of gene expression is also implicated in the epigenetic reprogramming driven by these viruses. Additionally, modification of host as well as pathogen transcribed RNA has been shown to influence stability and expression of transcripts in both cis- and trans- manner (Narayanan and Makino, 2013). Together, the mechanisms of DNA methylation, histone modifications, and non-coding RNA regulation form a complex network of epigenetic changes that enable oncogenic gamma herpesviruses to establish persistent infections, evade immune detection, and drive oncogenic transformation (Srivastava et al., 2023; Torne and Robertson, 2024). Understanding the mechanisms of epigenetic reprogramming by EBV and KSHV will not only sheds light on the fundamental processes of viral oncogenesis but also highlights potential therapeutic targets.

## Epigenetic histone marks induced on oncogenic *γ*-herpesvirus infection

Histones are lysine- and arginine-rich basic proteins that supercoil DNA around their exterior to form the nucleosome, which subsequently becomes chromatin through further superstructural organization (Millan-Zambrano et al., 2022). Generally classified into core (H2, H3, H4) and linker (H1, H5) histone groups, the canonical nucleosome consists of two H2-H3 dimers and a single H4 tetramer with DNA wrapped around this condensed hetero-octamer, which when combined with other hetero-octamers forms the chromatin superstructure (McGinty and Tan, 2015). Similar to the other proteins, histones are also amenable to various covalent posttranslational modifications which is fundamental to the cellular ability and necessity to regulate transcription enabling them to assemble into this superstructure and control access to the genome as their primary function (Millan-Zambrano et al., 2022; Chi et al., 2010).

The most common modifications to histones are generally associated with the activation or repression of gene transcription and include modifications such as the addition of methyl groups, acetyl groups (Zhu et al., 2021; Liu R. et al., 2020). More recently, citrullination- the Ca^2+^ − driven conversion of arginine into citrulline has been reported as a possible histone modification involved in carcinogenesis. However, the role of *γ*-herpesviruses in histone citrullination (or their impact on the phenomenon) remains poorly understood. As such, methylation and acetylation remain the most commonly studied modifications, with the net result of histone methylation on transcriptional access being dependent on the residue that gets methylated. H3K4 methylation is considered a transcriptional activation mark while H3K9 is considered a repressive mark (Miller and Grant, 2013). However, acetylation affects the chromatin superstructure in a way that allows enhanced transcriptional access to the genome via chromatin decondensation. Histone deacetylation, has the opposite effect where the chromatin superstructure returns to its normal tightly condensed organization, which subsequently represses transcription as compared to when the same residue is acetylated (Liu R. et al., 2020).

Within the realm of general viral infection, numerous studies have shown that viruses have evolved mechanisms through which they can deposit epigenetic marks like histone modifications near critical genes (e.g., CDKNA2, E-cadherin, IL-6, and RASSF1A) or transcriptional elements (promoter or enhancer regions) that allow them to survive, replicate, and produce new virions when reactivated (Pietropaolo et al., 2021; Flanagan, 2007; Li et al., 2005). More recently, evidence has emerged that some viruses engage in histone mimicry. Histone mimicry is generally defined as the incorporation of histone-like sequences in viral proteins that allow the virus to compete with canonical histone binding partners and promote viral gene expression (Tarakhovsky and Prinjha, 2018; Schaefer et al., 2013). Histone mimicry has been largely implicated as an epigenomic mechanism of SARS-CoV-2, which encodes a H3 histone mimic in its ORFs and has been shown to perturb post-translational modifications and promote chromatin compaction. One such example is ORF8 encoded by SARS-CoV-2, which mimic ARKS motif of H3 histone and interferes with host cell epigenome (Kee et al., 2022). In relation to oncogenic gamma herpesviruses, no antigens of EBV and KSHV are currently known to incorporate such sequences or perform such mimicking functions. In this section, we describe how EBV and KSHV affect the epigenetic landscape of their genomes through the manipulation of global and local patterns of specific histone modifications during infection, latency, and lytic reactivation.

### Epigenetic histone marks in KSHV infection

Critical facts regarding how KSHV actually infects its target cells remain to be understood, however, the involvement of various host cell receptors [DC-SIGN, Eph/integrin, and cysteine/glutamate antiporters (xCTs)], allows KSHV to have a very expansive cell tropism (Kerur et al., 2010; Veettil et al., 2008; TerBush et al., 2018). Currently known cell types that KSHV infects include B-cells, fibroblasts, monocytes, and endothelial cells that line blood and lymphatic vessels (Chakraborty et al., 2012). With regards to changes in epigenetic histone marks, the KSHV genome enters its target cell in an epigenetically naive state and subsequently undergoes significant changes to acquire various contrarian histone marks. These contrarian epigenetic reprogramming is often concomitant, following viral entry into the cell and includes both activating modifications (e.g., H3K4me3 and H3K36me3) as well as deactivating modifications (e.g., H3K27me3 and H3K9me3) (Srivastava et al., 2023; Dong and Weng, 2013; Campbell et al., 2020).

**Figure 1:**
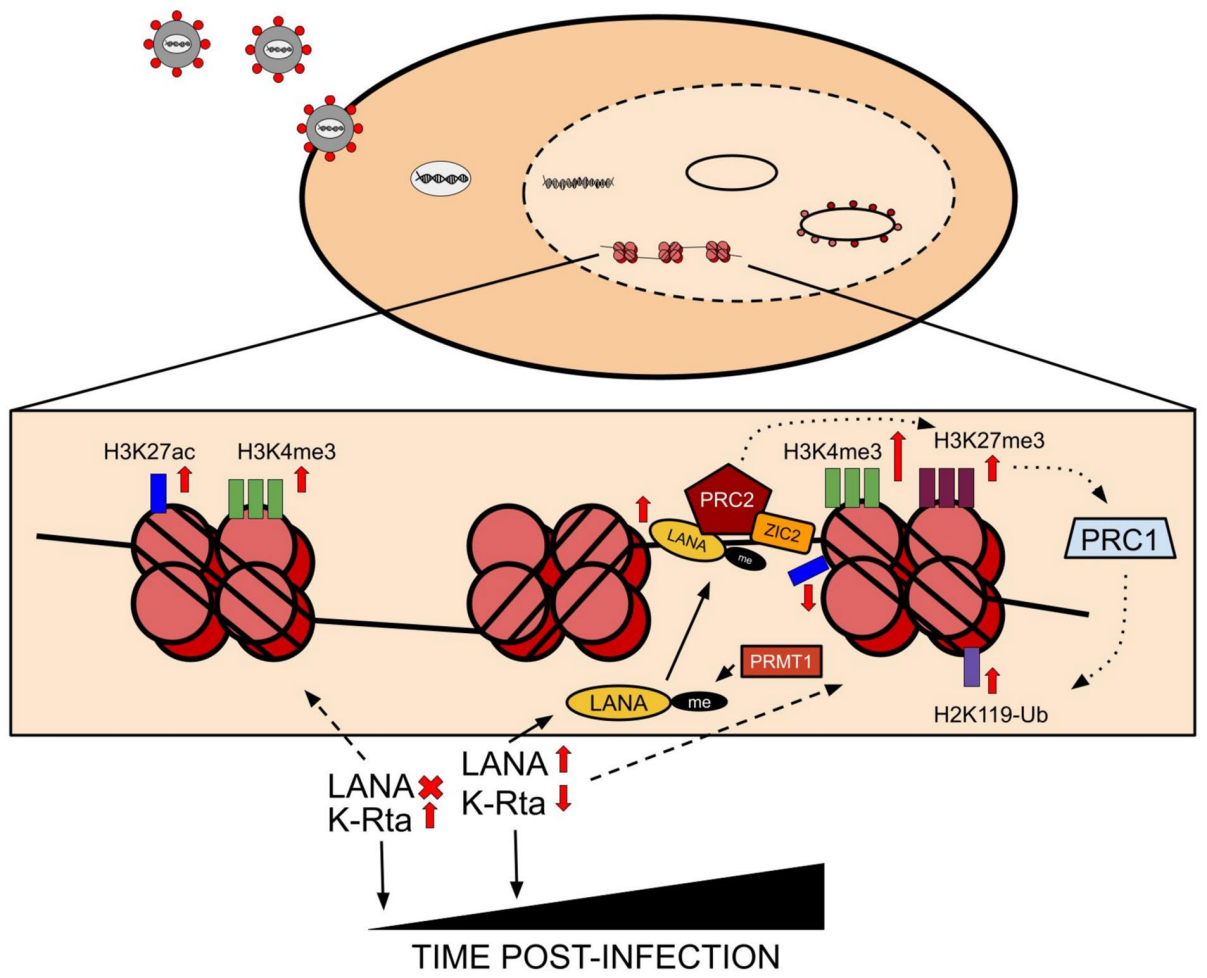
Following viral entry into the cell, the viral genome undergoes circularization and chromatinization. Subsequent induction of viral gene expression causes the initial expression of RTA, which results in the acquisition of activating histone marks such as H3K27ac and H3K4me3 across the chromatinized KSHV genome. Progressing from the initial stages of infection to establishing latency results in the induction of LANA expression, which causes major changes to occur to the histone mark patterns across the genome. Principally, LANA induction eventually suppresses RTA expression in cells, and the PRMT1-mediated methylation of LANA itself contributes to its efficient binding to chromatin. PRC2, the polycomb repressive complex 2, is attracted to LANA and is aided in stabilized binding to LANA by ZIC2, which aids in the induction of repressive mark deposition across the viral genome alongside an increase in the initial activating H3K4me3 mark. Principally however, LANA is responsible for leading to the deposition of repressive marks, and the PRC2/LANA/ZIC2 induced increase in H3K27me3 leads to the recruitment of PRC1 to the viral genome, which in turn deposits ubiquitinating repressive marks on the viral-bound histone 2A in the form of H2K119-Ub mark deposition.

The KSHV latent genome is known to associate with active and repressive epigenetic marks concomitantly with the deposition of repressive H3K9me3 and H3K27m3 marks and activating H3K4me3 across its genome (Figure 1; Toth et al., 2010). This concomitant, dual association with both marks has also been shown to be prominent in both primary and latent infection. However, there is a distinct pattern that results in the adoption of this concomitant state of epigenetic mark deposition. Following viral entry into the cells, the pre-chromatinization, epigenetically naive KSHV genome is fully exposed to host cell factors in an open chromatin landscape which induces the expression of master regulators like KSHV-encoded RTA, resulting in the deposition of transcriptionally activating histone marks such as H3K4me3 and H3K27ac on the viral genome (Figure 1; Toth et al., 2013). The activation of RTA transcription further attracts its host cell binding partners to the now pre-latent KSHV genome, such as RBP-Jκ (Lu et al., 2012). The presence of RBP-Jκ binding sites on the LANA promoter facilitates the transition of the KSHV genome from an epigenetically naive state to a latent, epigenetically mature state through deposition of specific histone marks that maintain and regulate the latent state of the virus (Uppal et al., 2014). Parallel to RBP-Jκ binding, naive, unmethylated CpG islands on the KSHV genome attract transcriptional repressors such as PRC1, whose catalytic subunit (RING1A/B ubiquitin ligases) can deposit repressive marks such as H2AK119 ubiquitination on the KSHV genome to promote latency (Toth et al., 2013). These initial changes occur within the first 72 h following primary KSHV infection, after which the KSHV epigenome becomes further enriched with H3K27me3 repressive histone marks (Toth et al., 2013). However, the dual adoption of repressive and activating marks continue, largely in part to the expression of LANA, which continues to aid in the deposition of H3K4me3 and H3K9me3, which mark viral genes for active transcription and transcriptional repression, respectively (Broussard and Damania, 2020). Prior to the full establishment of latency, arginine methylation of the LANA protein (mapped to arginine 20 of LANA) by PRMT1 (Protein arginine N-methyltransferase 1) contributes to its stabilization and subsequent LANA-mediated recruitment of hSET1 (histone-lysine N-methyltransferase SETD1A) and PRC2 (Polycomb repressive complex 2) to the KSHV genome. This results in further deposition of both activating (H3K4me3) and repressive (H3K27me3) histone marks, respectively on the genome, a prevalent condition on bivalent promoters (Toth et al., 2016; Campbell et al., 2012; Hu et al., 2014). LANA and vFLIP, both viral-encoded proteins, further enhance repressive mark deposition on the latent genome through the NF-kB pathway to induce the expression of a minimum number of viral genes necessary to maintain latency (Broussard and Damania, 2020; He et al., 2012). RTA, packaged in the virion and expressed immediately after initial infection, is subsequently regulated over time through numerous pathways to ensure that its latency-disrupting activity is minimized (Figure 1; Lan et al., 2005). For example, removal of the linear polyubiquitin chain of RTA by OTULIN prevents its nuclear localization and subsequent transactivation activity (Luan et al., 2024). From an epigenetic standpoint, the tethering of PRC2 to the RTA promoter by ZIC2 (Zic family member 2) and the binding of SIRT1, a class II histone deacetylase, form the two main routes through which the RTA promoter is in a constant state of hypermethylation via the deposition and maintenance of H3K27me3 and a reduction in H3K4 methylation to reduce promoter activity (Lyu et al., 2017; Hu et al., 2019). A brief overview of the dynamic histone mark deposition that occurs over time during KSHV *de novo* infection is shown in Figure 1. Epigenetic marks on histones are also prominent during KSHV lytic reactivation and are largely of the activating variety. Most notably, a reduction in symmetrical H4R3 methylation and increase in H3K4me3 promote an open chromatin landscape, which when combined with interactions between viral proteins and histone demethylases lead to reduced H3K27me3, which promote expansive lytic gene expression (Broussard and Damania, 2020; Strahan et al., 2017). One such example is association of KSHV-encoded ORF59 with PRMT5, where the interaction is believed to disrupt the H4R3 methylation and altering viral chromatin conformation (Strahan et al., 2017). A representative schematic showing interactions of KSHV-encoded antigens and histone modifications is shown in Figure 1.

### Epigenetic histone marks in EBV infected cells

EBV establishes itself in the host via primary epithelial cell infection and subsequent translocation of virions into B-cells, where they indefinitely remain quiescent following the establishment of latency. EBV latency is broadly characterized into three protein-expressing subtypes: latency III expresses the full suite of latent antigens and microRNAs, latency II (further subdivided into IIa and IIb) which expresses nuclear antigens alone (IIa) or membrane proteins alone (IIb), and latency I which expresses EBNA1 nuclear antigen only (Kelly et al., 2006; Chau et al., 2006; Kang and Kieff, 2015). The subdivision and resulting differential expression of viral genes in latency is largely perpetrated through the accumulation and dissociation of various histone marks on the latent EBV genome, in large part due to the accumulation of specific histone marks such as histone methylation near the three promoters that drive EBV latency: Cp, Wp, and Qp (Tierney et al., 2000; Tao et al., 1998; Fejer et al., 2008). The demethylation of lysine 4 and lysine 9 of histone H3 is considered as repressing modifications due to heterochromatin formation at these regulatory regions (Fejer et al., 2008; Chau and Lieberman, 2004).

Much like KSHV, EBV enters the cell in an epigenetically naive state and acquires various marks as it begins to establish latency (Buschle and Hammerschmidt, 2020). Following encapsidation and chromatinization of the EBV genome, acquisition of methylation marks across the major latent promoters - Cp, Wp, and Qp result in the beginning of differential gene expression of the latent genome through heavy suppression of most of the viral genome, albeit with leakage of lytic gene transcription in the pre-latent phase, and sometimes of late lytic genes in the early lytic phase (Murata et al., 2021; Rosemarie and Sugden, 2020). Similar to KSHV, there is concomitant acquisition of suppressive and activating histone marks on the EBV latent genome, most notably at the Qp promoter which has significant methylation potential but remains largely unmethylated throughout latency and the Cp promoter, which oscillates between hyper- and hypo-methylated depending on the latency type (Paulson and Speck, 1999; Robertson et al., 1995; Schaefer et al., 1997). Similarly, LMP expression driven by the LMP promoter is mediated by active histone marks such as H3K9Ac and H3K4me3, which are replaced by the repressive marks when LMP expression is silenced during specific latency subtypes III (Tempera and Lieberman, 2014; Day et al., 2007).

**Figure 2:**
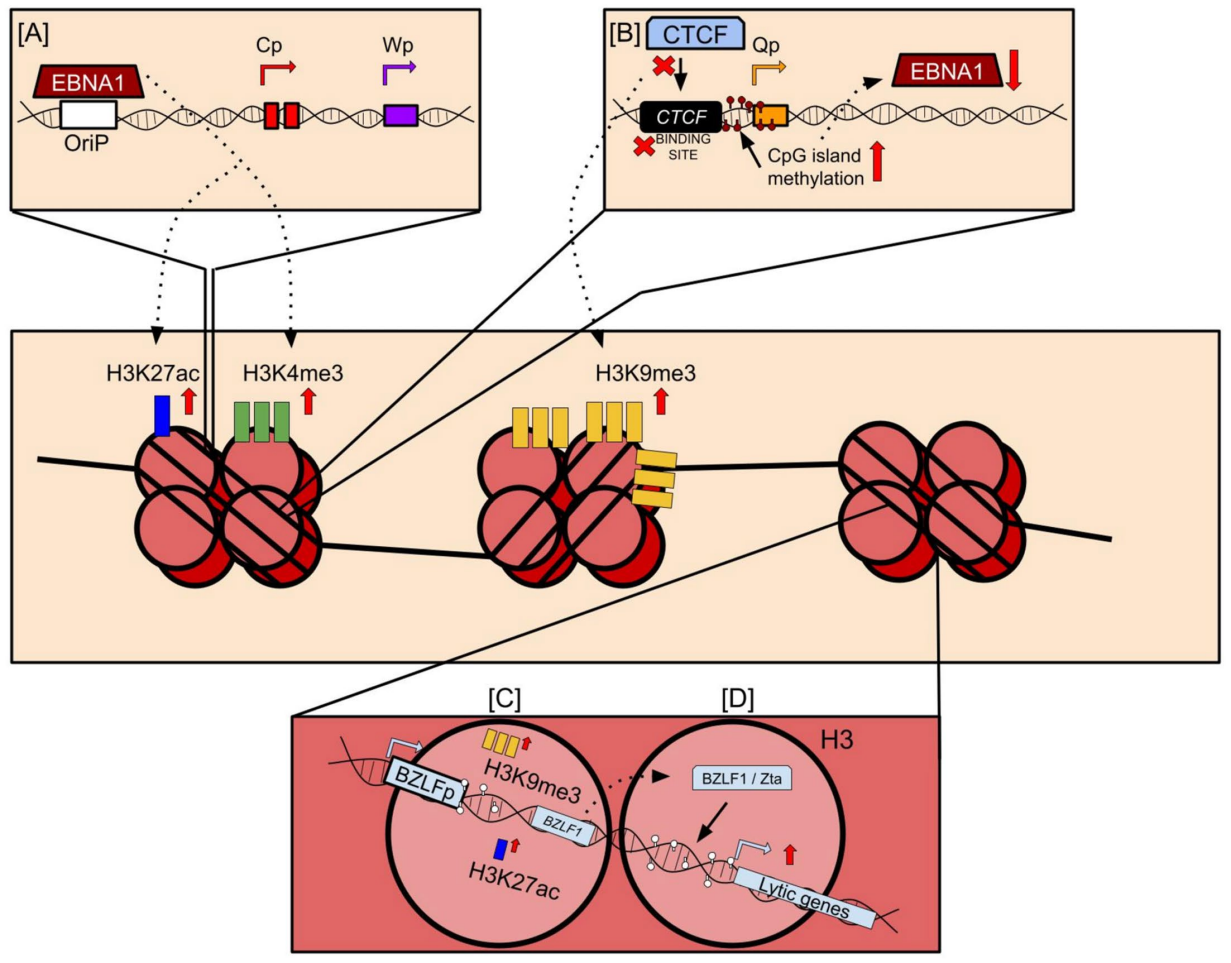
Histone mark deposition on the EBV viral genome. **(A)** EBNA1 binds to the origin of replication (OriP) and is critical for tethering the viral genome to the host genome. However, EBNA1 binding to the OriP also induces deposition of major activating histone marks such as H3K27ac and H3K4me3. **(B)** CTCF binding sites encoded proximal to promoters across the EBV genome bind CTCF and other host factors such as Cohesin to maintain the 3D structure of the genome. However, mutations at these sites or lack of CTCF binding to these sites may induce EBNA1 repression. This may occur through an increase in CpG island methylation proximal to promoters, or the deposition of repressive histone marks such as H3K9me3 onto the viral genome that represses viral gene expression. **(C)** CpG islands near promoters of lytic regulators such as BZLF1 are sparsely methylated, and instead acquire bivalent switch histone mark deposition through the acquisition of activating and repressive marks close to them. Following reactivation, repressive marks may be replaced with activating marks, leading to widespread expression of BZLF1 encoding the Zta protein. **(D)** Zta protein binds DNA motifs near CpG islands and induce downstream expression of viral genes during the lytic reactivation phase of the viral lifecycle.

A notable exception to histone mark deposition during EBV infection is the relative scarcity of the H3K27me3 mark on the EBV genome (Arvey et al., 2013). Considering the propensity of the same lysine residue (H3K27) to accommodate acetyl groups on its free amino tail, it is possible that the exclusion or scarcity of H3K27me3 offers a way to inhibit the complete suppression of the EBV genome and maintain consistent expression of EBNA1, which is necessary for EBV replication and is expressed across all latency types (Kang and Kieff, 2015). Some evidence to this theory comes from the mapping of histone marks deposited proximal to CTCF binding sites, which were heavily enriched for H3K27ac in all instances. Occurrence of multiple CTCF binding sites have been reported on EBV and KSHV genomes and considered to be retained in evolution for possible roles in both latency and reactivation. The presence of CTCF binding sites proximal to the Qp and LMP promoters allows molecules such as Cohesin to bind to the CTCF sites on the EBV genome and create distinct euchromatic and heterochromatic regions while also forming loops that brings regulatory elements together and perhaps even act as physical barriers to histone mark spreading (Figure 2; Tempera et al., 2010; Caruso et al., 2023). While the underlying reason for why this distinction is necessary remains to be understood, it is not beyond reach to speculate that incorporation of CTCF binding sites within the genome may be an evolutionarily incorporated mechanism necessary to prevent accidental methylation and suppression of critical antigens that are necessary to maintain latency and/or help the latent virus escape host immunosurveillance.

Concomitant to its importance to latency, latent histone mark deposition is also the principal causal agent for EBV lytic suppression along with CpG island methylation (Murata et al., 2021). When undergoing lytic reactivation, the epigenetic profile of the latent genome is rapidly altered to accommodate the enhanced activity necessary for lytic gene expression and virion assembly. Principally, the promoter of EBV latency-to-lytic switch regulator Zta, transcribed by BZLF1, forms a bivalent switch by incorporating both activating and repressive marks on itself, although a more expansive presence of repressive marks is necessary to suppress lytic activity during latency (Figure 2; Murata et al., 2012). This repressive activity is enhanced or maintained by well-characterized histone marks such as H3K27me3 and H3K9me3 (Ramasubramanyan et al., 2012b; Woellmer et al., 2012; Imai et al., 2014), which is easier to demethylate following lytic stimuli exposure compared to CpG island methylation and requires a multi-step demethylation, glycosylase reaction, and DNA synthesis processes (Wu and Zhang, 2017). Following lytic reactivation, the histone marks associated with transcriptional repression are removed in an effort to promote transcription of the lytic elements of the viral genome. Figure 2 provides a brief overview of the major histone marks deposited onto the EBV viral genome and the specific mechanisms that induce this deposition. Despite their evolutionary differences and mechanisms of perpetrating histone mark depositions, EBV and KSHV contribute to similar epigenetic profiles that are largely representative of a hypermethylated viral genome for the purpose of evading host cell immunosurveillance that allows these viruses to persist in its hosts indefinitely. However, the deposition of these marks on histones is just one of numerous strategies evolved with these viruses that enable them to both persist in hosts and contribute to their associated malignancies. A representative figure for interactions of EBV-encoded antigens and histone modification is shown in Figure 2.

### DNA modification marks due to oncogenic *γ*-herpesvirus infection

Similar to the histone modification, EBV and KSHV, employ DNA methylation as an alternate or concurrent mechanism to alter host gene expression, which is required for promotion of viral persistence as well as oncogenesis (Chen et al., 2013; Journo et al., 2021). DNA methylation involves addition of methyl groups to cytosine residues in CpG dinucleotides, resulting in differential transcription of the associated genes (Jin et al., 2011). EBV manipulates this process by upregulating host DNA methyltransferases (DNMTs) which results in hypermethylation and subsequent silencing of target genes, generally tumor suppressors or those involved in host defense system (Scott, 2017). By silencing these genes, EBV facilitates uncontrolled cell proliferation and enhances the invasive potential of infected cells (Low et al., 2023). Similarly, KSHV utilizes its encoded proteins, such as LANA, to promote the hypermethylation of promoters of tumor suppressor genes by interacting with DNMTs (Shamay et al., 2006). Nevertheless, the viral genome is also modified through methylation to induce suppression of viral encoded genes to the extent which is conducive for latency. This strategic reprogramming of the host’s epigenome allows these viruses to create a cellular environment that supports viral persistence, evades immune detection, and drives the transformation of infected cells (Toth et al., 2010).

### DNA methylations by EBV and KSHV viruses

EBV induces host DNA methylation changes that lead to silencing tumor suppressor genes involved in cell cycle control, signaling pathways, apoptosis, invasion, and migration (Mui et al., 2017; Soliman et al., 2021). The EBV proteins LMP1, LMP2A, and EBNA3C recruit DNA methyltransferases (DNMTs) to promoters and induce hypermethylation (Tsai et al., 2002; Hino et al., 2009; Zhang et al., 2019). For EBV, DNA methylation regulates the expression of viral latent and lytic genes and relies on extensive DNA methylation of its genome to maintain latency, with promoters of lytic genes being heavily methylated (Farrell, 2019). In contrast, the latent EBV promoters are largely unmethylated (Fernandez et al., 2009; Bergbauer et al., 2010). During lytic reactivation, the BZLF1 transcription factor encoded by EBV preferentially binds to methylated DNA motifs (Lang et al., 2019) (meZREs) to activate early lytic genes, despite the overall high methylation of the viral genome (Bhende et al., 2004; Dickerson et al., 2009). The EBV oncoprotein LMP1 methylates the promoter of lysine-specific demethylase 2b (KDM2B), which demethylates histone 3 at the lysine 4 (H3K4me3) site, leading to transcriptional silencing (Yang et al., 2017). EBV causes a high frequency of methylation of DNA in the host genome, e.g., several tumor suppressor genes [APC (adenomatous polyposis coli protein), PTEN (Phosphatidylinositol 3,4,5-triphosphate 3-phosphate and dual-specificity protein phosphatase), and RASSF1A (Ras associated domain-containing protein 1A)] and cell adhesion molecules (THBS1 and E-cadherin) (Okabe et al., 2020). The EBV protein EBNA2 interacts with cellular transcription factors and promotes the expression of genes involved in cell proliferation, contributing to development of EBV-associated lymphomas (Yin et al., 2019). EBV utilizes DNA methylation as a key epigenetic mechanism to control the expression of its latent genes. The promoters within EBV genome like Wp, Cp, and Qp are subjected to differential methylation patterns during different latency programs (Paulson and Speck, 1999; Guo and Gewurz, 2022). This allows the virus to switch between latency types and minimize its expression to evade immune detection (Paulson and Speck, 1999). EBV activates DNA methyltransferase activity by increasing the expression of DNMT1, DNMT3a, and DNMT3b (Song et al., 2022). The latent membrane protein 1 (LMP1) is responsible for altering replication (32, the DNA methyltransferase activity) (Song et al., 2022). EBNA1 tethers the latent viral episomes to host chromosomes during cell division, ensuring the viral genome is transmitted to daughter cells (Frappier, 2012). The EBV lytic activator Zta can selectively bind to methylated viral DNA, enabling the establishment and reactivation of methylated viral genomes (Ramasubramanyan et al., 2012a).

When the EBV genome is methylated at the Cp promoter, transcription initiates at the Qp promoter to produce only the EBNA1 protein, without the other EBNA genes. This switch from Cp to Qp occurs as B-cells differentiate from proliferating centroblasts to resting memory B-cells (Chen et al., 2013; Takacs et al., 2009). Concurrently, methylation of LMP1 and LMP2 promoters leads to their stable repression. EBV-encoded antigens can also target DNA methyltransferases and methyl-binding proteins and directly bind to methylated DNA to alter the host DNA methylation machinery (Hsieh, 1999; Dawson and Kouzarides, 2012). Methylation of the BZLF1 and BRLF1 promoters also controls the switch between EBV latency and lytic replication (Feederle et al., 2000). The EBV latent antigen EBNA3C upregulates the methyltransferase METTL14, which cooperates with EBNA3C to promote cell growth and proliferation (Lang et al., 2019). BZLF1, a highly expressed EBV gene, represses METTL3 expression at the transcript levels by binding to its promoter region. A reduced expression of METLL3 is directly related to the reduced m6A modification of the host gene KLF4 transcripts. Moreover, the knockdown of m6A reader YTHDF2 increases KLF4 transcripts stability (Dai et al., 2021). METTL3 promotes the production of EBV progeny virions and the expression of late viral lytic protein. It also enhances the expression of the EBV latent antigen EBNA2 (Yanagi et al., 2022; Zheng et al., 2021). The m6A reader YTHDF1 promotes binding of RNA degradation complexes to the mRNAs of EBV lytic genes BZLF1 and BRLF1, suppressing EBV infection and replication (Woellmer and Hammerschmidt, 2013). DNA methylation is also necessary for Zta-dependent binding, transcriptional activation, and lytic gene expression. However, DNA methylation is absent at transcriptionally active latency promoters and other protected sites like OriP and Qp. DNA methylation represses Cp in type I latency, resulting in EBNA2 and EBNA3 silencing (Woellmer and Hammerschmidt, 2013; Karlsson et al., 2008; Kalla et al., 2012).

KSHV also induces differential methylation of host DNA in infected cells (Kuss-Duerkop et al., 2018; Journo et al., 2018). The KSHV protein LANA recruits DNMT3A to chromatin, leading to hypermethylation of genes involved in cell cycle, signaling, and metastasis (Shamay et al., 2006). Other KSHV proteins, like vIRF1 and vIL6, can also modulate DNA methylation (Wu et al., 2014; Li et al., 2020). KSHV-encoded proteins like LANA can directly or indirectly alter the cellular epigenome. LANA interacts with chromatin-modifying enzymes like EZH2 and recruits DNA methyltransferases to repress host genes. KSHV miRNAs can also affect DNA methylation patterns in the viral and cellular genomes (Pyakurel et al., 2006). The KSHV genome undergoes extensive DNA methylation, with global methylation of the viral episome except for the latency-associated locus (Lopes et al., 2022; Darst et al., 2013). This DNA methylation acts as a reinforcer of viral gene expression inhibition caused by repressive histone marks (Gunther and Grundhoff, 2010). KSHV also modulates host cellular DNA methyltransferases (DNMTs) to induce DNA methylation on specific cellular promoters, leading to their repression (Flanagan, 2007; Li et al., 2005). For example, the KSHV-encoded LANA protein can recruit the host DNMT3A to the promoters of host-encoded genes (Ballestas and Kaye, 2011). The KSHV protein LANA interacts with the DNA methyltransferase DNMT3a, activating it and recruiting it to the host chromatin DNA to methylate the promoter of the down-regulated gene cadherin 13 (H-cadherin) (Shamay et al., 2006). These epigenetic alterations contribute to KSHV-driven oncogenesis. KSHV directly interacts with and recruits the *de novo* DNA methyltransferase DNMT3a to cellular chromatin, leading to the repression of approximately 80 host-encoded genes. KSHV also interacts with the DNA methyl-binding protein MeCP2 and the histone methyltransferase SUV39H1, enabling epigenetic gene regulation (Shamay et al., 2006).

**Figure 3:**
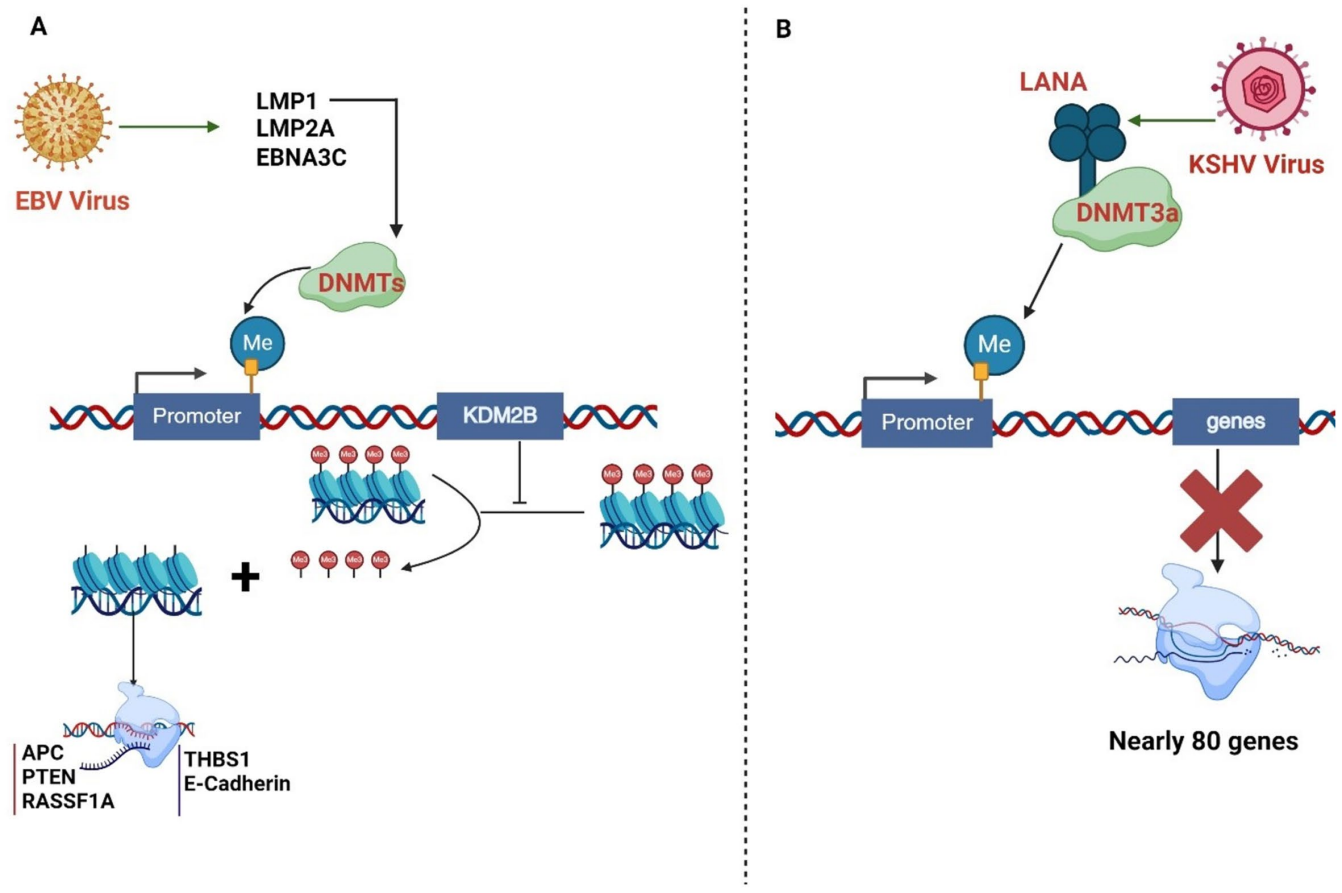
**(A)** EBV proteins LMP1, LMP2A, and EBNA3C recruit DNA methyltransferases (DNMTs) to promoters, inducing hypermethylation. LMP1 activates DNMTs, promoting hypermethylation of various gene promoters, including tumor suppressor genes. KDM2B, a histone demethylase, can be silenced by DNMT-mediated promoter methylation, affecting its gene expression regulation function. **(B)**. The KSHV protein LANA interacts with the DNA methyltransferase DNMT3a, activating it and recruiting it to the host chromatin DNA to methylate the promoter. These epigenetic alterations contribute to KSHV-driven oncogenesis. KSHV directly interacts with and recruits the *de novo* DNA methyltransferase DNMT3a to cellular chromatin, resulting in repression of approximately 80 cellular genes.

Latency-associated nuclear antigen (LANA) encoded by KSHV binds to and recruits the de novo DNA methyltransferase DNMT3a and the methyl-CpG binding protein MeCP2, leading to repression of lytic cycle gene expression (Shamay et al., 2006). DNMT3a and 3b also mediate CpG methylation and transcriptional repression of the MHV68 ORF50 promoter during latency. KSHV genome is also extensively methylated, where ORF50/RTA promoter being highly methylated during latency (Pantry and Medveczky, 2009). KSHV also exhibits repressive histone modifications like H3K9me3 and H3K27me3 on its genome, which are associated with latency (Gunther et al., 2019). The viral LANA protein recruits chromatin modifiers like PRC1/2 (Polycomb repressive complex 1/ 2) and SUV39H1 to regulate viral and cellular gene expression (Toth et al., 2016). KSHV and EBV are known to utilize the host DNA sensor IFI16 to maintain their latency (Cesarman et al., 1995). During lytic reactivation, KSHV selectively degrades IFI16 through polyubiquitination and proteasomal degradation (Roy et al., 2016). This allows the virus to relieve the transcriptional repression exerted by IFI16 on the viral lytic genes, enabling full-fledged lytic replication (Roy et al., 2016). DNA methylation does not occur at constitutively active latency promoters like the LANA promoter but instead occurs at several transcriptionally inactive regions in the latent infection conditions. KSHV has complex histone modification patterns during latent infection, with bivalent control of gene expression at the ORF50 promoter (Toth et al., 2010). Figure 3 is a represent action of the intricate interactions of EBV and KSHV which leads to modulation of the epigenome.

### Epigenetic regulation of viral and cellular transcripts by oncogenic gamma herpesviruses

In addition to the direct modification of DNA and DNA bound histones, gene expression is also regulated epigenetically at the transcript level (Gibney and Nolan, 2010). This epigenetic regulation can be mediated in a cis- as well as trans- manner. Over 100 different types of RNA epigenetic modifications have been identified, many with context-dependent functions (Liu and Pan, 2015). The cis-acting elements are specific RNA sequences or structures that regulate various processes such as splicing, stability, localization, and translation of the RNA molecules. They function intrinsically by interacting with the RNA itself or nearby molecules within the same RNA molecule (Elcheva and Spiegelman, 2020). Trans-acting RNA regulatory factors operate externally by binding to other molecules, including proteins, DNA, or other RNA molecules (Elcheva and Spiegelman, 2020; Carrier et al., 2020; Ali Syeda et al., 2020). The well-established cis- and trans- acting elements working at the transcript level for epigenetic regulation include but is not limited to microRNAs (miRNA), long non-coding RNA (lncRNA), RNA interference (RNAi), RNA nucleoside modifications (e.g., m6A, m5C etc.) and RNA binding proteins (RBPs) (Elcheva and Spiegelman, 2020; Carrier et al., 2020). In this section, we discuss the major pathways involved in epigenetic regulation at the transcript level during EBV/KSHV infection.

### Epigenetic regulation of transcripts by trans-acting factors in oncogenic *γ*-herpesviruses

The MicroRNAs (miRNAs) are small non-coding RNAs of approximately 22 nucleotides in length, which play a role in regulation of gene expression in higher organisms (Ratti et al., 2020). They regulate the target mRNAs by base-pairing to the untranslated regions (UTRs) of the target mRNAs, thereby leading to mRNA cleavage or translation inhibition (Ratti et al., 2020). These miRNAs play an important regulatory role in cell physiology by negatively controlling gene expression and are involved in regulation of critical pathways such as cell differentiation, proliferation, cell cycle, angiogenesis, and autophagy, making them particularly interesting in the study of cancer (Macfarlane and Murphy, 2010). Noncoding RNAs (ncRNAs) are also, now recognized as factors in controlling genes and can act as either cancer-promoting or cancer-suppressing in various types of malignancies associated with oncogenic virus infection. EBV was reported as one of the earliest oncogenic virus to encode such RNAs and disruption of coding RNAs is highly evident in both EBV and KSHV-associated cancers (Notarte et al., 2021; Zhang et al., 2020; Pfeffer et al., 2004). Several studies have shown changes in both B cell and epithelial cell miRNA expression patterns following EBV or KSHV infection. EBV-related epithelial tumors, type II and III latency programs exhibit distinct miRNA expression profiles (Cameron et al., 2008; Cai et al., 2006). In the case of NPC, where most tumors exhibit type II latency, there is a preference for increased expression of BHRF1 miRNAs activated by the viral oncogene LMP1 (Lo et al., 2007). The increase in BHRF1 miRNAs can be linked to demethylation-mediated activation of the BHRF1 gene locus (Li et al., 2017). The transcription of BART miRNAs, produced from the BART-encoded noncoding RNA, is a common characteristic observed during both epithelial and B cell infections with EBV (Li et al., 2017). BART miRNAs are abundant in tumors associated with EBV and have been shown to target various genes, both cellular and viral (Qiu et al., 2011). The increase in BART miRNAs has been linked to the disruption of promoter methylation through different pathways (Kim et al., 2011).

The KSHV genome is reported to encode 13 pre-miRNAs which are processed by the cellular machinery to yield 25 mature miRNAs (Guo et al., 2017; Qin et al., 2017; Gottwein et al., 2011). The majority of known KSHV-miRNAs are located in the latent locus and expressed during the latent phase of virus infection (Samols et al., 2005). The Kaposin promoter regulates clusters of KSHV miRNA genes during viral latency. Many KSHV pre-miRNA genes appear to be intergenic, positioned between the Kaposin gene sequence and the open reading frame (ORF) 71 of the KSHV genome (Umbach and Cullen, 2010). KSHV-miR-K12-10 and KSHV-miR-K12-12 are expressed at higher levels during the lytic phase and located within ORF and 3′UTR of the Kaposin gene (Lin et al., 2010). The rest of the KSHV-encoded miRNAs are expressed strictly during the latent phase from an approximately 4-kb noncoding sequence located between Kaposin and ORF71 (Qiu et al., 2011). MiR-K9-5p and miR-K7-5p, can also target the KSHV-encoded RTA. KSHV miRNAs, miR-K12-1, −3 and − 4-3p target Casp3, which blocks apoptosis. Inhibition of these miRNAs with specific oligonucleotides directed to the seed regions enhances apoptosis of KSHV-infected cells (Suffert et al., 2011). Furthermore, miR-K12-11 targets IKK epsilon (IKK), which regulates interferon signaling and promotes latency of the KSHV virus (Arias et al., 2014). DNA methyl-transferase-1 (DNMT1) also methylates the RTA promoter, maintaining viral latency. V-miR-K12-4-5p controls DNMT1 activity by repressing the retinoblastoma-like protein 2 (RBL2) in favor of viral latency (Lu et al., 2010). KSHV-encoded miRNAs are now reported to modulate several pathways including angiogenesis, cell cycle, cell migration, and adhesion which are critical to KSHV dissemination and pathogenesis (Samols et al., 2007; Gallaher et al., 2013). Several studies independently reported that KSHV miRNAs impact the functions of immune effector cells and thus alter the secretion pattern of many cytokines including IL-6, IL-8, and IL-10 (Boss and Renne, 2010; Nachmani et al., 2009; Qin et al., 2010). Besides encoding its own miRNAs, KSHV infection has been shown to significantly alter expression of many cellular miRNAs. These miRNAs, in turn, regulate KSHV entry, replication, pathogenesis, and immune evasion (Choi et al., 2015; Qin et al., 2014). Interestingly, HIV-1 viral protein R (Vpr) inhibits KSHV lytic replication cycle via upregulation of miR-942-5p and associated signaling activities that targets IκBα (Yan et al., 2016). Vpr also targets Notch1 and thus inhibits NF-κB signaling, which is required to support KSHV lytic replication (Yan et al., 2018). KSHV-K15, a viral oncoprotein has been shown to induce cell migration and angiogenesis via upregulation of cellular miR-21 and miR-31. Similarly, downregulation of miR-221/miR-222 cluster in KSHV infection has been reported to enhance the migration pattern of endothelial cells (Qin et al., 2014). Thus, KSHV-induced cellular miRNAs may function in conjugation with the KSHV-encoded miRNAs to promote latency and tumorigenesis.

### Epigenetic regulation of transcripts by cis-acting factors during oncogenic *γ*-herpesviruses infection

Chemical modifications on mRNA were first discovered in the 1970s using poly(A) tail-based purification techniques that ensured mRNA preparations were pure enough and excluded from contamination from other RNA types. A notable example of such regulation is the discovery of reversible 6-methyladenosine (m6A) modifications in mRNAs, along with the key enzymes that regulate them: writers, erasers, and readers. Additionally, evidence has been accumulating demonstrating a regulatory role for modifications such as pseudouridine, 5-methylcytidine (m5C), or 1-methyladenosine (m1A) in mRNAs (Shi et al., 2020). Modifications such as m6A are reported to be present in all types of RNA such as mRNA, tRNA, rRNA or even in mitochondrial transcripts, while others such as m1A are more prominently present in tRNA and rRNA (Shi et al., 2020). Pseudouridine (*Ψ*), one of the most abundant RNA modification in cells, is produced through the enzyme-mediated isomerization of uridine (Zhao and He, 2015). While 5-methylcytidine (m5C) is mainly recognized for its significant role as an epigenetic mark in DNA, it was also found in early RNA methylation studies (Gao and Fang, 2021). Initial mapping of m5C in RNA used bisulfite sequencing technology adapted from DNA, identifying about 10,000 m5C sites in mRNA (Gao and Fang, 2021). Furthermore, most RNA modifications occur on the base moiety, but methylation on the ribose 2′ hydroxyl group to form 2′OMe has been detected on all four ribonucleosides in various RNA classes (Nachtergaele and He, 2017).

Viral infections cause significant changes in cellular gene expression. Some of these changes occur as the host responds to the infection, while others result from viral hijacking of the host cell machinery to promote or inhibit specific gene expression programs that impact their lifecycle (Locatelli and Faure-Dupuy, 2023). For instance, several viruses manipulate cellular mechanisms to aid in the export of viral RNA from the nucleus, increase the stability of viral RNA, enhance the translation of specific messages, and facilitate the packaging and release of viral particles (Rajendren and Karijolich, 2022). One way viruses achieve this hijacking is by manipulating post-transcriptional RNA modifications (Rajendren and Karijolich, 2022). These RNA modifications not only affect the viral lifecycle but also modulate the host’s immune response (Rajendren and Karijolich, 2022). They act as key chemical markers for distinguishing self from non-self and alter the expression of antiviral molecules (Karandashov et al., 2024). Consequently, RNA modifications play a crucial role in both the progression and restriction of viral infections. Below we provide some examples of prominent cis-acting modifications of RNA, which are capable of regulating epigenetic activities in cis-acting manner.

### N6-methyl-6-adenosine modification

**Figure 4:**
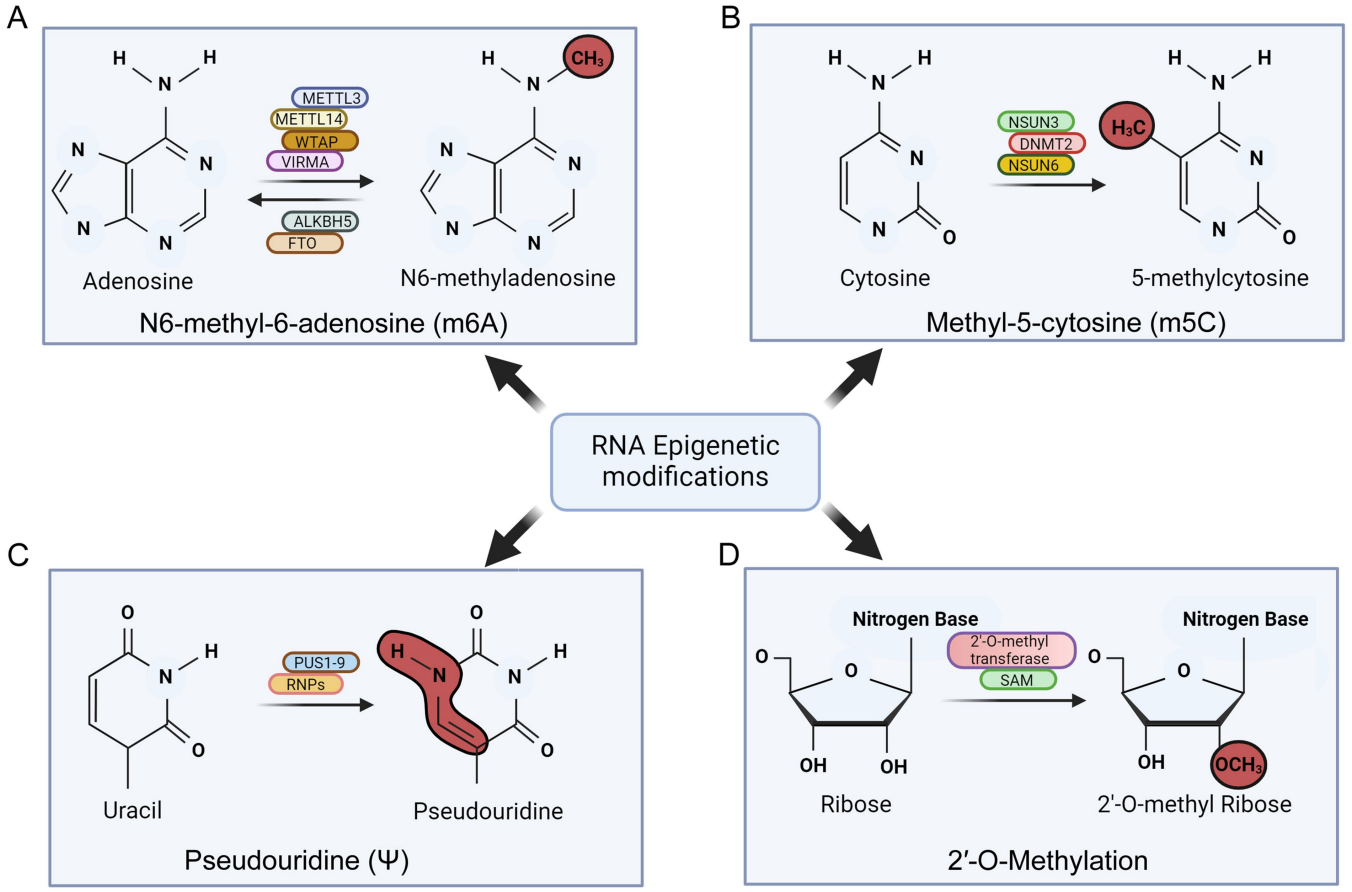
Schematic showing various post-transcriptional modifications of RNA in oncogenic *γ*-herpesvirus infection. The figure summarizes the major factors involved in mediating these modifications. **(A)** N6-methyl-6-adenosine (m6A) modification of RNA. **(B)** Methyl-5-cytosine (m5C) modification of RNA. **(C)** Psudouridine (*ψ*) modification of RNA and **(D)** 2’-O-methylation of RNA.

The N6-methyladenosine (m6A) modification in RNA molecules involves the addition of a methyl group at the N6 position of the adenosine nucleotide, making it the highest common internal modification in mRNA (Liu et al., 2023). This modification is catalyzed by enzymes known as “writers” and occurs co-transcriptionally in the nucleus (Cai et al., 2022). The methyltransferase complex responsible for m6A deposition on mRNA is formed by METTL3 and METTL14, using S-adenosylmethionine (SAM) as a substrate (Wang et al., 2016). Additionally, the METTL3/METTL14 complex is associated with the WT1-associate protein (WTAP), which is crucial for substrate targeting (Wang et al., 2016). Vir-like m6A methyltransferase associated protein (VIRMA) represent another example responsible for m6A RNA methylation (Figure 4A; Li et al., 2022). “Reader” proteins recognize m6A-modified mRNA molecules to execute the function of the modification (Wang et al., 2016). The YTH-domain family (YTH) is the best-characterized group of m6A reader proteins, involved in regulating various aspects of mRNA metabolism, including alternative splicing, nuclear export, translation efficiency, degradation, and subcellular localization (He and He, 2021). Furthermore, enzymes called “erasers,” such as the ALKB homolog 5 RNA demethylase (ALKBH5) and alpha-ketoglutarate-dependent dioxygenase (FTO), can reverse this modification through demethylation reactions (Rau et al., 2019).

EBV-encoded BZLF1 can directly influence m6A modification of RNA by influencing expression of writer enzyme METTL3 (Dai et al., 2021). A low m6A modification of EBV-encoded transcript and hence its stability in down-regulated condition of METTL3 can provide can provide a strategy of immune evasion by EBV (Yanagi et al., 2022). Additionally, EBV infection was shown to induce hypomethylation of TLR9 mRNA, affecting its stability and potentially aiding in viral latency (Zhang et al., 2024). Deleting METTL3 in B cells reduces the expression of viral lytic proteins and decreases the production of progeny virions, indicating METTL3’s role in the EBV replicative cycle (Figure 4A; Yanagi et al., 2022).

Previously, we demonstrated that EBNA3C activated transcription of METTL14, and directly interacted with METTL14 to promote its stability (Lang et al., 2019). Recently, we also reported that during the initial phase of lytic reactivation, the virus overcomes the cellular innate immune response by modulating the methylation marks on some cellular mRNAs (Bose et al., 2023).

KSHV infection is also reported to exhibit extensive m6A methylation on viral and cellular mRNAs during both lytic and latent replication (Manners et al., 2023; Tan et al., 2018). KSHV-encoded antigens interact with the host cell’s m6A modification machinery to enhance viral gene expression and evade host immune responses (Macveigh-Fierro et al., 2020). KSHV-encoded ORF57 protein has been shown to recruit m6A methyltransferases to modify viral RNA, promoting efficient RNA processing, nuclear export, and translation (Macveigh-Fierro et al., 2020). Additionally, m6A modifications facilitate the switch between latent and lytic phases of the viral life cycle, critical for KSHV persistence and pathogenesis (Tan et al., 2018; Macveigh-Fierro et al., 2020). Enzymes responsible for this modification play crucial roles in KSHV infection. Published data suggest that the reader protein YTHDF2 suppress KSHV infection by promoting de-adenylation and degradation of viral transcripts (Hesser et al., 2018).

### Pseudouridine (Ψ) modification

Pseudouridine (Ψ) is the most common internal RNA modification in transfer RNA (tRNA) and ribosomal RNA (rRNA). This irreversible modification occurs through both RNA-dependent and RNA-independent manner. The RNA-dependent pathway involves H/ACA small nuclear ribonucleoproteins (sRNPs) using a guide RNA (gRNA) complementary to the target sequences (Spenkuch et al., 2014). The gRNA hybridizes with the target sequences, forming a secondary structure recognized by the sRNPs, which then catalyze the conversion of uracil to pseudouridine (Yu and Meier, 2014). The RNA-independent pathway involves pseudouridine synthase enzymes (PUS), which recognize secondary structures in RNA molecules containing uridine targets (Rintala-Dempsey and Kothe, 2017). These enzymes modify tRNA (PUS 2/3/4/6/9), small nucleolar RNA (snRNA) (PUS 1/7), rRNA (PUS 5/7), and mRNA (PUS 1/2/3/4/6/7/9). Notably, no reader proteins for pseudouridine have been identified so far (Figure 4B; Soto et al., 2022). While pseudouridylation is well-documented in tRNA and rRNA, its role in mRNA is still unclear. Studies have shown that pseudouridylation in pre-mRNA can affect alternative splicing by changing the affinity of RNA-binding proteins or altering secondary structures within pre-mRNAs (Pederiva et al., 2023). Pseudouridylation may also stabilize RNA by modifying its structure and promoting a more rigid backbone through base-pairing (Borchardt et al., 2020). Pseudouridylation in mRNAs can be recognized by a methionine-specific tRNA synthetase (MetRS), for regulatory translation purposes. This recognition may decrease the binding of antisense RNA molecules, thereby enhancing translation efficiency (Levi and Arava, 2021). KSHV was found to modulate the pseudouridylation pathway by altering the activities of pseudouridinase PUS1 and PUS7 responsible for incorporation of pseudouridines during lytic reactivation of KSHV (Atari et al., 2022). EBV-encoded non-coding RNA1 and 2 (EBER1 and EBER2) are known to be modified with pseudouridines to enhance their stability, which is essential during lytic reactivation (Figure 4B; Henry et al., 2022).

### Methyl-5-cytosine and 2′-O-methylation modifcations

The m5C modification is facilitated by RNA m5C methyltransferases, part of the Rossman fold–containing enzyme superfamily, using S-adenosyl-l-methionine (SAM) as a methyl donor (ref). Identified m5C-specific methyltransferases include NSUN1–7 in humans and the DNA methyltransferase (DNMT) homolog DNMT2 (Zhang Y. et al., 2022). Among the NSUN family, NSUN2 is one of the most thoroughly researched and serves as a primary m5C writer (Zhang Y. et al., 2022). The function of m5C methylation is regulated by m5C-binding proteins like ALYREF and YBX1, which bind specifically to m5C sites in RNA (Figure 4C; Zhang Y. et al., 2022). Recent research suggests that m5C cytosine methylation impacts various cellular processes, including nuclear RNA export, mRNA translation, cell cycle regulation, stem cell differentiation and proliferation, development, and cancer (Xiong et al., 2024). Bisulfite sequencing showed that about 95% of EBV encoded EBER1 molecules have a single methylated cytosine (Henry et al., 2020). The RNA methyltransferase NSUN2 was identified as the enzyme responsible for this specific methylation (Henry et al., 2020). Notably, removal of NSUN2 resulted in the loss of m5C modification, and an increased level of EBER1. Thus this modification adversely affects the stability of viral-encoded lncRNA (Henry et al., 2020).

Of the two primary mechanisms for bulk eukaryotic mRNA export that are well-understood is through the recognition of C(5)-methylation marks on mRNAs. The NXF1-dependent bulk mRNA export pathway, where an NXF1-NXT1 heterodimer associates with mRNA through the TREX-1/2 complex. TREX-1 includes components like ALYREF/THOC4, UAP56, CIP29, PDIP3, ZC11A, UIF, and the THO subcomplex (THOC1/2/3/5/6/7), with ALYREF serving as an adaptor. This adaptor protein specifically recognizes the C(5)-methylation marks on mRNAs and regulates their nuclear to cytoplasmic export (Yang et al., 2017). THOC4/ALYREF are vital for export of KSHV intronless mRNAs and the production of infectious viruses through recruitment of the TREX complex (Figure 4C).

2′-O-methylation (Nm, where N denotes any nucleotide) is a co- or post-transcriptional RNA modification, involving the addition of a methyl group (− CH3) to the 2′ hydroxyl (− OH) of the ribose. This modification can occur on any nucleotide and is a highly conserved and abundant feature found in transfer RNA (tRNA), ribosomal RNA (rRNA), and small nuclear RNA (snRNA) (Dimitrova et al., 2019). Nm is also present at various sites in messenger RNA (mRNA) and at the 3′ end of small non-coding RNAs (sncRNAs), such as microRNAs (miRNAs) and small interfering RNAs (siRNAs) in plants, on Ago2-loaded si- and miRNAs in flies, and on PIWI-interacting RNAs (piRNAs) in animals (Xiong and Zhang, 2023). Nm is known to impact RNA in multiple ways. It increases hydrophobicity, protects against nuclease degradation, stabilizes helical structures, and affects interactions with proteins or other RNAs. For instance, Nm enhances the thermodynamic stability of RNA base pairs and stabilizes A-form RNA duplexes. Additionally, Nm modifications can disrupt RNA tertiary structures and inhibit RNA-protein interactions through steric hindrance or by altering hydrogen bonding (Figure 4D; Dimitrova et al., 2019).

Role of 2′-O-methylation has also been studied in the context of EBV and KSHV infection. In EBV, 2′-O-methylation plays a vital role in viral gene expression and immune evasion. EBV RNAs undergo 2′-O-methylation to prevent detection by host innate immune sensors like RIG-I, thereby avoiding immune responses that would otherwise hinder viral replication (Pan et al., 2022). Similarly, KSHV exploits 2′-O-methylation to stabilize its transcripts and ensure efficient protein synthesis, crucial for both latent and lytic phases of its life cycle (Atari et al., 2022). These modifications also aid in immune evasion, allowing the virus to persist in the host by reducing immunogenicity of its RNAs. Both viruses utilize host methyltransferases, such as Fibrillarin and other members of the small nucleolar RNA (snoRNA) family, to introduce 2′-O-methylation marks (Hutzinger et al., 2009). Understanding the role of 2′-O-methylation in EBV and KSHV infection will unveil new insights into viral pathogenesis and highlights potential therapeutic targets. A schematic for various RNA modification and the associated factors is provided in Figure 4.

## Conclusion

The study of histone and DNA methylation marks in oncogenic *γ*-herpesvirus infections has elucidated significant mechanisms through which these viruses manipulate host cellular epigenetic landscapes to promote their persistence and oncogenic potential. Both Epstein–Barr virus (EBV) and Kaposi’s Sarcoma-associated herpesvirus (KSHV) exploit epigenetic modifications to orchestrate their life cycles, evading host immune surveillance and promoting oncogenesis. Both EBV and KSHV demonstrates a dynamic interplay between activating and repressive histone marks during their infection cycles. The employed strategy of histone modification allows these viruses to establish and maintain latency, and disruption can lead to lytic reactivation. The deposition of both activating marks like H3K4me3 and repressive marks such as H3K27me3 reflects a finely tuned balance necessary for viral persistence and periodic activation. Furthermore, KSHV’s ability to recruit host chromatin-modifying enzymes and alter DNA methylation profiles underscores its sophisticated mechanisms for evading immune detection and promoting malignancy. EBV’s strategic use of DNA methylation to silence tumor suppressor genes and modulate its own latent gene expression further illustrates its ability to influence host cellular mechanisms to favor its persistence and oncogenic potential.

Both viruses exemplify the critical role of epigenetic regulation in viral pathogenesis and the development of associated cancers. By altering histone and DNA methylation patterns, EBV and KSHV create a cellular environment conducive to their survival and replication while also contributing to tumorigenesis. Future research focusing on the precise molecular mechanisms underlying these epigenetic modifications may offer new insights into therapeutic strategies aimed at disrupting these viral processes and treating related malignancies. Understanding the roles of histone and DNA modifications during infection with these viruses will be helpful for developing targeted interventions and advancing our knowledge of virus-host interactions in the context of oncogenic diseases.

The interplay of EBV and KSHV with host cellular machinery is profoundly intricate, particularly at the transcript level where epigenetic regulation plays a crucial role. Both EBV and KSHV exploit a variety of mechanisms to modulate host RNA, thereby influencing their own lifecycle and the cellular environment to favor viral persistence and pathogenesis. MicroRNAs (miRNAs) and long non-coding RNAs (lncRNAs) are pivotal in regulation of gene expression. EBV and KSHV have evolved sophisticated strategies to manipulate these RNA species. EBV-encoded miRNAs, including BHRF1 and BART, disrupt cellular gene expression patterns, contributing to oncogenesis and immune evasion. Similarly, KSHV-encoded miRNAs are integral to viral latency and pathogenesis, influencing cellular processes such as apoptosis, angiogenesis, and immune modulation. The interaction between viral and host miRNAs further complicates the regulatory landscape, highlighting the complex relationship between the virus and host cellular machinery.

The cis-acting factors are also crucial in regulating gene expression during infection with these viruses. Modifications of RNA, such as m6A, pseudouridine (*Ψ*), and 5-methylcytosine (m5C), are critical to the regulation RNA stability, translation, and splicing. EBV and KSHV manipulate these modifications to enhance their replication and evade host immune responses. For instance, m6A modifications in EBV and KSHV RNAs facilitate various aspects of the viral lifecycle, from gene expression to immune evasion. Similarly, pseudouridine and 2′-O-methylation modifications contribute to the stability and translation efficiency of viral RNAs, further aiding in the persistence and pathogenesis of these viruses.

### Future directions

Understanding the mechanisms by which gamma herpesviruses modulate epigenetics opens new avenues for therapeutic interventions. Targeting specific RNA modifications or the interactions between viral and host epigenomes at the level of DNA, histone or RNA may provide novel strategies for controlling viral infections and associated malignancies. Future research could focus on elucidating the precise roles of these modifications during the viral lifecycle, immune modulation, as well as exploring potential therapeutic agents that can disrupt these interactions. In summary, the ability of oncogenic gamma herpesviruses to alter the host-pathogen epigenome through the wide array of mechanisms underscores their adaptability and pathogenic potential. Continued research into these processes will be crucial for developing targeted therapies and improving our understanding of viral oncogenesis.
